# Peripheral Muscle Function and Body Composition in People With Cystic Fibrosis on Elexacaftor/Tezacaftor/Ivacaftor: A Cross‐Sectional Single‐Centre Study

**DOI:** 10.1002/ppul.71044

**Published:** 2025-03-12

**Authors:** Lauren J. Clayton, Anthony I. Shepherd, Jo Corbett, Mathieu Gruet, Gary Connett, Mark Allenby, Julian Legg, Thomas Daniels, Don S. Urquhart, Zoe L. Saynor

**Affiliations:** ^1^ School of Psychology, Sport and Health Science, Faculty of Science and Health University of Portsmouth Portsmouth UK; ^2^ University Hospital Southampton NHS Foundation Trust Southampton UK; ^3^ Université de Toulon Toulon France; ^4^ National Institute for Health and Care Research, Southampton Biomedical Research Centre Southampton UK; ^5^ Department of Paediatric Respiratory and Sleep Medicine Royal Hospital for Children and Young People Edinburgh Scotland UK; ^6^ Department of Child Life and Health University of Edinburgh Scotland UK; ^7^ School of Health Sciences, Faculty of Environmental and Life Sciences University of Southampton Southampton UK

**Keywords:** cystic fibrosis transmembrane conductance regulator, modulator, muscle strength, obesity, skeletal muscle

## Abstract

**Background:**

People with cystic fibrosis (pwCF) often have multifactorial peripheral muscle abnormalities attributed to, for example, malnutrition, steroid use, altered redox balance and, potentially, CF‐specific intrinsic alterations. Malnutrition in CF now includes an increasing prevalence of overweight and obesity, particularly in those receiving CF transmembrane conductance regulator (CFTR) modulator therapy (CFTRm). We aimed to characterise peripheral muscle function and body composition in pwCF on Elexacaftor/Tezacaftor/Ivacaftor (ETI) CFTRm, compared to healthy controls.

**Methods:**

Fifteen pwCF on ETI, and 15 healthy age‐ and sex‐matched controls (CON), underwent whole‐body dual‐energy X‐ray absorptiometry scans, and a comprehensive evaluation of peripheral muscle function. Tests included quadriceps maximal isometric force measurement, an intermittent isometric quadriceps fatiguing protocol, handgrip strength dynamometry, squat jump height assessment, and 1‐min sit‐to‐stand testing.

**Results:**

No significant differences in quadriceps maximal isometric force (CON: 181.60 ± 92.90 Nm vs. CF: 146.15 ± 52.48 Nm, *p* = 0.21, *d* = 0.47), handgrip strength (CON: 34 ± 15 kg vs. CF: 31 ± 11 kg, *p* = 0.62, *d* = 0.18), peripheral muscle endurance, fatigue, or power were observed between the groups. Moreover, no significant differences in whole‐body, trunk or limb lean mass, fat‐free mass, fat mass, or whole‐body bone mineral density were evident.

**Conclusion:**

Comparable peripheral muscle mass and function has been demonstrated in pwCF on ETI, albeit a group with good lung function. Research is needed to confirm these findings longitudinally in pwCF, including those with more severe lung disease, who are less physically active, and have less optimal nutrition and exercise support.

## 1 Introduction

Advancements in cystic fibrosis (CF) treatment, particularly with highly‐effective CF transmembrane conductance regulator (CFTR) modulator therapies (HEMT), have greatly improved the life prospects of many people with CF (pwCF). However, this extended lifespan introduces new concerns about co‐morbidities and long‐term health [1]. Pre‐HEMT, pwCF have shown reduced bone mineral density (BMD) [2] and peripheral skeletal muscle dysfunction, including reduced muscle mass, strength [3], endurance, and increased fatigability [4]. The impact of HEMT, particularly newer combinations like Elexacaftor/Tezacaftor/Ivacaftor (ETI), on these muscle‐related issues remains unclear.

Several mechanisms contribute to peripheral skeletal muscle dysfunction in CF, including those common in chronic respiratory disease (e.g., atrophy, malnutrition, chronic inflammation, oxidative stress, hypoxaemia, and steroid use [4]), and a potential CF‐specific, CFTR‐related intrinsic muscle defect [5, 6]. CFTR proteins are expressed in the sarcoplasmic reticulum [5, 7], and pre‐HEMT research shows mitochondrial metabolic abnormalities [8], such as a heightened calcium (Ca^2+^) response in CFTR deficient myotubes [7]. HEMT may therefore enhance skeletal muscle function, through both direct and indirect effects, potentially modulating Ca^2+^ homeostasis [9], and muscle oxidative capacity [10], as recent studies suggest [11].

HEMT has been associated with increases in body mass index (BMI) and fat mass (FM) [12, 13], while fat‐free mass (FFM) remains stable [12], contributing to a rising prevalence of obesity, including normal weight obesity (NWO) [13] that brings new health concerns in this population. Proposed mechanisms include improved appetite, reduced resting energy expenditure, gut inflammation, and reduced fat malabsorption [14]. FFM depletion [12] has been associated with worsened lung function, systemic inflammation, and higher rates of pulmonary infection [15]. Since BMI alone does not differentiate between FM and FFM, dual‐energy X‐ray absorptiometry (DEXA) is a more accurate body composition assessment tool [13].

This study therefore aimed to: (1) evaluate peripheral skeletal muscle function, including strength, power, endurance, and fatigability; (2) characterise body composition; and (3) examine relationships between body composition and peripheral skeletal muscle function, in pwCF on ETI compared to healthy controls (CON). We hypothesised that FFM, BMD, peripheral muscle strength, power, endurance and fatigue would be significantly lower and FM higher in pwCF on ETI compared to CON.

## 2 Materials and Methods

### 2.1 Ethical Approval

This UK based, single centre, cross‐sectional study, represents a subset analysis from the Understand‐CF study. The study was pre‐registered on ClincalTrials.gov (NCT05857709) and received favourable ethical opinion from the South Central—Berkshire B NHS Research Ethics Committee and Health Research Authority (22/SC/0168). Fully‐informed written consent was provided by adult participants and caregivers if participants were < 18 years, who themselves provided assent. Data collection was conducted at the University of Portsmouth (UK). Participants refrained from alcohol, caffeine and intense exercise for 24‐h before testing, and arrived ≥ 2‐h post‐prandial.

### 2.2 Study Participants

An a priori power calculation was performed, using quadriceps maximal voluntary contraction (MVC) data [16]. For 95% power and *p* < 0.05 (two‐tailed), nine participants per group were required to detect a significant difference. To account for dropouts, the target sample size was 15 pwCF.

Fifteen pwCF (seven children/adolescents and eight adults; Table 1) were recruited from the University Hospital Southampton NHS Foundation Trust, along with 15 age‐ and sex‐matched healthy CON (seven children/adolescents and eight adults; Table 1) from the local area. Inclusion criteria for pwCF included a CF diagnosis based on clinical features and a previous abnormal sweat test (sweat chloride > 60 mmol·L^−1^ > 100 mg sweat), stable on ETI, ≥ 10 years of age, and no recent (within 2 weeks) increase in symptoms or weight loss. Exclusion criteria included ineligibility for ETI, unstable co‐morbid asthma (daily pulmonary function variability > 20%), or vasoactive medication use. Control participants were selected by age and sex, free from any chronic disease, not smokers, not pregnant, not contraindicated to exhaustive exercise, and willing/able to understand and cooperate with the study.

**Table 1:** Baseline anthropometric, pulmonary function and physical activity data for participant with cystic fibrosis and healthy age‐ and sex‐matched control participants upon initiation into the study.

Variable	CON (*n* = 15)	PwCF (*n* = 15)	*p* value	ES
Months on ETI	—	25.8 ± 9.1	—	—
Genotype (*n*)	—	∆F508 Homozygote, *n* = 7; ∆F508 Heterozygote, *n* = 7; Other, *n* = 1		
Pancreatic insufficient (*n*)	—	15	—	—
CF‐related diabetes (*n*)	—	3	—	—
CF‐related liver disease (*n*)	—	5	—	—
Sex (F/M)	4/11	4/11	—	—
Age (year)	24.3 (14.9–43.4)	24.4 (14.0–46.4)	0.94	0.02
Stature (m)	1.69 ± 0.15	1.67 ± 0.14	0.72	0.13
Weight (kg)	66.2 ± 19.6	63.9 ± 17.2	0.74	0.12
BMI (kg·m^2^)	22.8 ± 5.4	22.6 ± 3.4	0.88	0.06
BMI *z*‐score^a^	−0.36 ± 0.78	0.38 ± 0.63	0.07	1.05
MUAC (cm)	27.93 ± 4.91	27.87 ± 3.83	0.97	0.01
FVC (L)	4.20 ± 1.41	3.98 ± 1.31	0.67	0.16
FVC (% predicted^c^)	96.0 ± 12.0	95.9 ± 15.9	0.98	0.01
FVC z‐score	–0.31 ± 0.88	–0.36 ± 1.29	0.91	0.04
FEV_1_ (L)	3.30 ± 1.11	2.94 ± 1.08	0.37	0.33
FEV_1_ (% predicted^c^)	91.1 ± 9.8	85.7 ± 23.0	0.41	0.31
FEV_1_ *z*‐score	−0.71 ± 0.71	−1.09 ± 1.76	0.45	0.28
FEF_25__%−75%_ (%)	3.0 ± 1.1	2.6 ± 1.4	0.41	0.31
FEF_25__%−75%_ (% predicted^c^)	81.6 ± 20.6	72.6 ± 33.6	0.38	0.32
FEF_25%−75%_ *z*‐score	−0.84 ± 0.82	−1.27 ± 1.68	0.38	0.33
PA parameters^b^	—	—	—	—
Average acceleration (mg)	38.75 (34.4–961.33)	28.41 (20.59–44.53)	0.11	0.33
Intensity gradient	−2.36 ± 0.33	−2.42 ± 0.25	0.63	0.29

*Note:* Parametric values are presented as means ± SD, where data were analysed using independent *t*‐test and effect sizes were estimated using Cohen's *d*. Effect size, 0.2 = small effect, 0.5 = medium effect, 0.8 = large effect. Non‐parametric values are presented as median (interquartile range), where data were analysed using Mann−Whitney *U*‐test and effect sizes were estimated using Rosenthal's *r*. Effect size, 0.2 = weak effect, 0.4 = moderate effect, 0.6 = strong effect.

Abbreviations: BMI, body mass index; ES, effect size; ETI, Elexacaftor/Tezacaftor/Ivacaftor; F, female; FEF_25%−75%_, forced mid‐expiratory flow; FVC, forced vital capacity; FEV_1_, forced expiratory volume in 1‐s; M, male; MUAC, mid‐upper arm circumference.

^a^ indicates a group where *n* = 7, consisting of paediatric individuals, for whom *z*‐scores are available;

^b^ Indicates a group where *n* = 12;

^c^ According to Quanjer et al. [17].

### 2.3 Participant Characteristics

Clinical characteristics of pwCF were recorded from their most recent clinical assessment (Table 1). Pulmonary function (Table 1) was assessed via spirometry using a turbine flow‐meter system (COSMED BMI Ltd, Rome, Italy), in accordance with current guidelines. Absolute values were expressed as percentages of predicted normal and *z*‐scores, based on current reference values. Height was measured to the nearest 0.01 m (Seca 213 stadiometer, UK), and body mass to the nearest 0.01 kg (Seca 770 scales, UK). BMI (with *z*‐scores) was calculated. Mid‐upper arm circumference (MUAC) was measured at the mid‐point of the acromion of scapula and olecranon of ulna of the relaxed left arm, with the average of three measurements recorded to the nearest 0.1 cm. Pubertal staging was self‐assessed according to pubic hair classification in participants < 18 years. Free‐living physical activity (PA) was measured using a GENEActiv triaxial accelerometer (Activinsights Ltd, Cambridge, UK) in accordance with Clayton et al. [18].

### 2.4 Outcome Measures

#### 2.4.1 Peripheral Muscle Function

A comprehensive battery of tests was used to evaluate lower body muscle strength, power, endurance, and fatigability, and forearm strength, following recommended guidance [19] and previous work in CF [20]. Specifically, participants completed a 1‐min sit‐to‐stand (STS) test [21, 22], with 3 squat jumps pre‐ and immediately post‐STS testing [23], followed by a handgrip strength (HGS) test [24, 25]. After a 30‐min recovery, the 1‐min STS test and squat jumps were repeated [22]. Following a further 30‐min recovery, a quadriceps intermittent fatigue test which is reliable [26] and has previously been used in pwCF [20], was undertaken.

##### 2.4.1.1 Peak Quadriceps Torque

Torque produced during quadriceps MVC was measured on the dominant leg, using an isokinetic dynamometer (HUMAC NORM, Stoughton, USA), following global recommendations [19]. Participants were seated with both knees at 90° flexion and hips at 130° (see Supporting Information Material, Supporting Information S1: Figure 3). Participants performed two baseline MVC manoeuvres, separated by 30‐s rest [19, 20].

##### 2.4.1.2 Quadriceps Endurance and Fatigue

Knee extensor endurance and fatigue were assessed using established methods [26], consistent with prior CF studies [20]. The best peak torque of the two baseline MVC manoeuvres was used to calculate individualised work rates for the fatiguing protocol. Participants subsequently performed sets of 10 intermittent submaximal isometric contractions (5‐s on: 5‐s off), starting at 10% of baseline MVC, and increasing by 10% increments until exhaustion (see Supporting Information Material, Supporting Information S1: Figure 3) [20]. Exhaustion was defined as the inability to maintain the target force for >2.5 consecutive seconds. Visual feedback (target and produced force) was provided on‐screen throughout. After each set, immediately after exhaustion, and following 10‐min of recovery, participants performed two MVC manoeuvres seperateed by a 30‐s rest.

##### 2.4.1.3 HGS

HGS, a measure of muscle strength and nutritional status [25], previously used in CF [24, 25], was assessed using a grip dynamometer (Takei, 5401, Japan). In line with recent recommendations [19], participants were seated with 90° elbow angle flexion and the hand in a neutral position, performing three maximal efforts each lasting 5‐s, with the highest value recorded.

##### 2.4.1.4 Integrated Test of Muscle Function

The 1‐min STS test was conducted with a 40.4 cm fixed height chair (without armrests) placed against a wall [19]. Participants stood up with straightened legs and sat down with clear contact on the chair, as many times as possible in 1‐min, keeping hands on hips. Participants set their own pace, with standardised encouragement, and were notified when 15‐s remained. The best of two tests, separated by 30‐min, was recorded. No significant learning effect was evident.

The 1‐min STS induces significant fatigue [27] and indicators, like changes in a single STS performance or squat jump height (SJ_H_), are sensitive to the development of muscle fatigue [28], which can be captured with smartphone applications such as the “MyJump2” application. The “MyJump2” application has proven valid and reliable [28, 29] for calculating jump height via high‐speed video [30]. Maximal power output of the lower limb extensor muscles was measured with three squat jumps, pre‐ and immediately post‐ the 1‐min STS (see Supporting Information Material, Supporting Information S1: Figure 4). Participants began with feet shoulder‐width apart, hands on hips, and knees at 90° flexion. They jumped as high as possible while keeping hands on hips. SJ_H_ was recorded using the My Jump 2 application (Apple Inc. USA) on an iPhone 11, with 120‐fps recording mode video. The phone was placed 1.5 m from the participant's feet. SJ_H_ was calculated, by selecting the take‐off frame (first frame where both feet were off the ground) and landing frame (first frame where at least one foot was touching the ground) on the video, using the following equation: SJ_H_ (m) = flight time (s)^2^ × 1.22625 [31].

#### 2.4.2 Body Composition

##### 2.4.2.1 DEXA

Whole‐body and regional (arms, legs, trunk) BMD, FFM, lean mass (LM) and FM were obtained using a whole‐body DEXA scan (Hologic, Vertec, UK; Table 2). Participants were scanned in loose clothes, without metal or shoes, and in a supine position. Whole‐body composition analysis followed operator guidelines (Hologic Whole‐Body DXA Reference Database software, Hologic, Vertec, UK), manipulating segmental lines. Horizontal lines were positioned below the mandible and above the iliac crest. A lower pelvic divider line was positioned between the top of the femur and the femoral head. Vertical lines were positioned between the head of the humerus and scapula at the glenoid fossa, left and right of the spine, between the hand and leg on each side and between the legs.

**Table 2:** Peak exercise variables for the 1‐min sit‐to‐stand data for participants with CF and age‐ and sex‐matched healthy controls.

Variable	CON (*n* = 15)	PwCF (*n* = 15)	*p* value	ES
1‐min STS				
Repetitions (reps·min^−1^)	43 (35−54)	37 (28−56)	0.37	0.17
Repetitions x weight (kg·min^−1^)	2938 ± 1078	2497 ± 676	0.19	0.49
Power_STS_ (W)	296 ± 133	243 ± 61	0.18	0.51
Peak heart rate^a^ (beats·min^−1^)	143 ± 21	141 ± 18	0.83	0.08
Peak SpO_2_ (%)	98 (97−98)	97 (96−97)	< 0.01*	0.62
∆SpO_2_ (%)	0 (0−0)	1 (0−1)	< 0.01*	0.53
Peak Borg muscle fatigue (a.u)	13 ± 1	13 ± 2	0.93	0.03
Peak Borg breathlessness (a.u)	12 (11−13)	12 (10−14)	0.95	0.02
Squat jump				
SJ_H_ (cm)	26.93 ± 11.62	22.43 ± 6.71	0.21	0.47
Decline in SJ_H_ (cm)	0.32 ± 2.19	0.11 ± 1.85	0.78	0.10

*Note:* Parametric values are presented as means ± SD, where data were analysed using independent *t*‐test and effect sizes were estimated using Cohen's *d*. Effect size, 0.2 = small effect, 0.5 = medium effect, 0.8 = large effect. Non‐parametric values are presented as median (interquartile range), where data were analysed using Mann−Whitney *U*‐test and effect sizes were estimated using Rosenthal's *r*. Effect size, 0.2 = weak effect, 0.4 = moderate effect, 0.6=strong effect.

Abbreviations: CON, control group; ES, effect size; PwCF, people with cystic fibrosis; SpO2, transcutaneous arterial oxygen saturation; STS, sit‐to‐stand.

^a^ Indicates a group where *n* = 13.

* Indicates significant difference (*p* < 0.05).

#### 2.4.3 Statistical Analysis

Statistical analyses were conducted using IBM SPSS Statistics (version 27.0, IBM Chicago, IL). Normality was checked with the Shapiro−Wilk test. Normally distributed data were analysed using independent samples *t*‐tests and presented as means ± standard deviations (SD). Effect sizes (ES) were estimated using Cohen's *d*: small (*d* = 0.2), medium (*d* = 0.5), and large (*d* = 0.8). Pearson's correlations were used to explore relationships between variables, classified as negligible (*r* = 0.00–0.10), weak (*r* = 0.10–0.39) moderate (*r* = 0.40–0.69), strong (*r* = 0.70–0.89) and very strong (*r* = 0.90–1.00). For non‐normally distributed data, Mann−Whitney *U*‐tests were used, presented as medians and interquartile ranges (25th and 75th percentiles), with *ES* estimated using Rosenthal's *r:* weak (*r* = 0.2), moderate (*r* = 0.4), and strong (*r* = 0.6). Significance was set at *p* < 0.05.

## 3 Results

### 3.1 Participant Characteristics

DEXA imaging and muscle function tests were conducted within 6 ± 14‐days. One participant was lost to follow‐up and did not undergo DEXA scanning. One participant did not consent to provide pubertal maturation data. Pubertal maturity for participants < 18 years old was: pre‐pubertal (CF, *n* = 2; CON, *n* = 2), circumpubertal (CF, *n* = 3; CON, *n* = 3), and post‐pubertal (CF, *n* = 1; CON, *n* = 0). The full de‐identified data set has been made freely available on the University repository (DOI: 10.17029/66ed94a3‐f968‐44ce‐8af5‐484bb1d6890f).

### 3.2 Peak Quadriceps Torque

**Figure 1:**
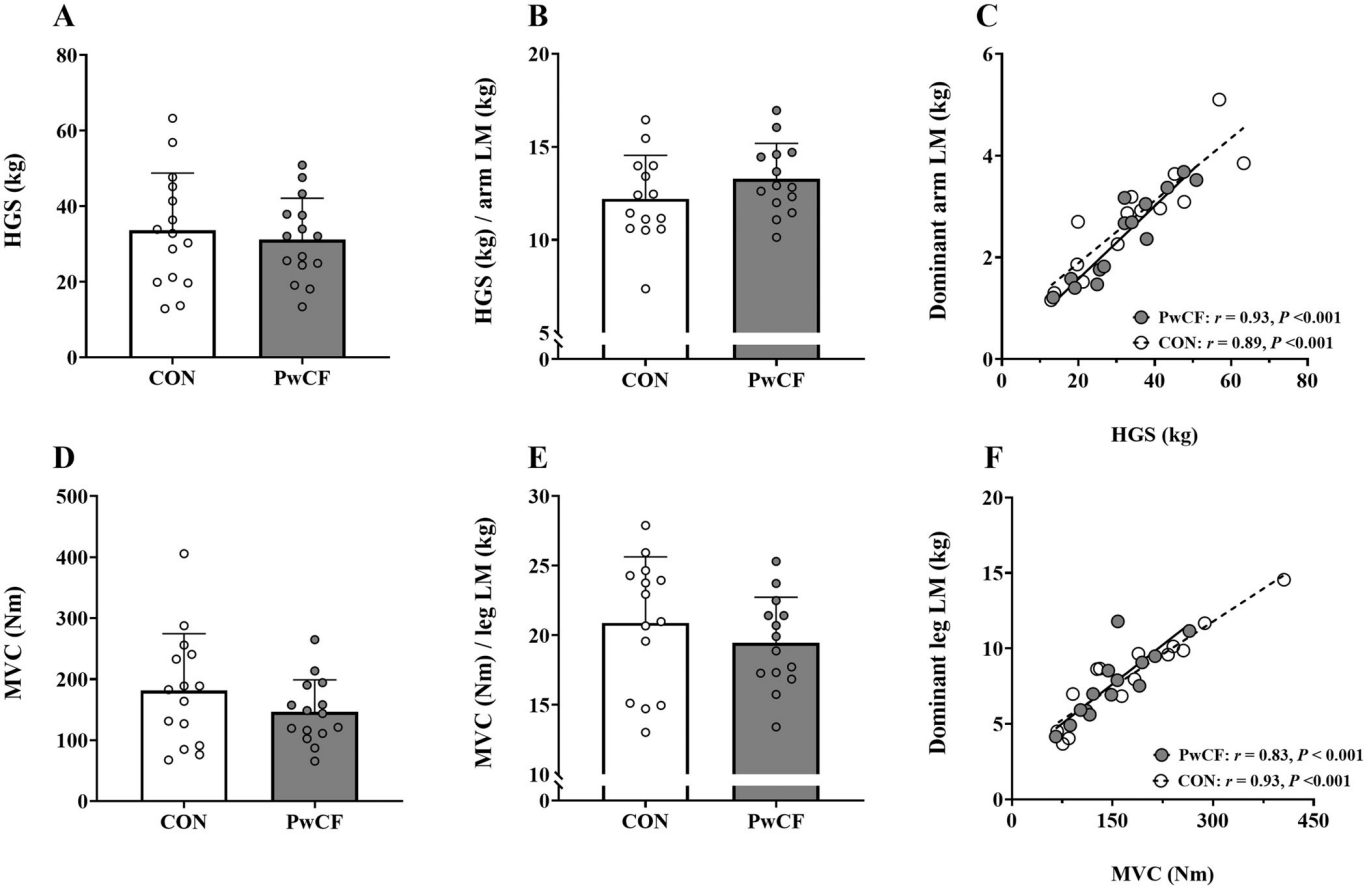
Parameters for handgrip strength and maximal voluntary contraction in pwCF and healthy control participants. N.B. Parametric values are presented as means ± SD, where data were analysed using independent t‐test. Handgrip strength test (HGS) (A), HGS normalized to dominant arm lean mass (LM) (B), correlation between HGS and dominant arm LM (C), quadriceps maximal voluntary isometric force (MVC) (D), MVC normalised to dominant leg LM (E), correlation between MVC and dominant leg LM (F), in CON (white) and pwCF (gray).

Peak torque during the MVC (CON: 181.60 ± 92.90 Nm vs. CF: 146.15 ± 52.48 Nm, *p* = 0.21, *d* = 0.47), and peak torque when expressed relative to dominant leg LM (CON: 20.89 ± 4.74 Nm/kg vs. CF: 19.44 ± 3.29 Nm/kg, *p* = 0.36, *d* = 0.50), were not different between groups (Figure 1).

### 3.3 HGS

HGS (CON: 34 ± 15 kg vs. CF: 31 ± 11 kg, *p* = 0.62, *d* = 0.18) and HGS, when expressed relative to dominant arm LM (CON: 12.21 ± 2.33 kg vs. CF: 13.27 ± 1.92 kg, *p* = 0.20, *d* = 0.47), was not different between groups (Figure 1).

### 3.4 Quadriceps Endurance and Fatigue

**Figure 2:**
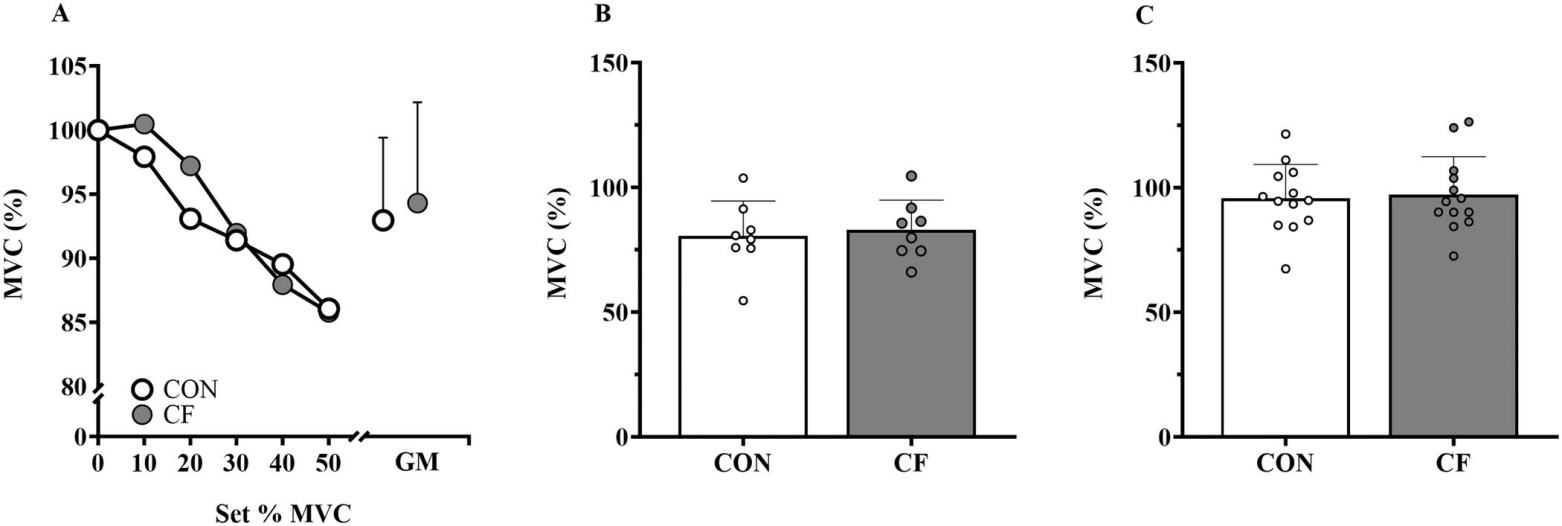
Parameters for decline in maximal voluntary contraction during intermittent fatiguing task in pwCF and healthy control participants. N.B. % of baseline maximum voluntary contraction (MVC) at baseline (0), after each set of the fatiguing task (10, 20, 30, 40, 50) and grand means (GM) ± SD for full sets completed between matched groups (A), % of baseline MVC immediately after exhaustion (Exh) (B) and post‐10‐min (P10) of recovery (C). Pre, 10, 20, 30, *n* = 14; 40, *n* = 12; 50 *n* = 10; Exh, *n* = 8, P10, *n* = 13).

Total number of submaximal contractions was not different between CON and CF (62 ± 14 vs. 57 ± 11, respectively, *p* = 0.27, *d* = 0.43). There were no differences in the decline in MVC during the fatiguing task (Figure 2A), immediately after exhaustion (Figure 2B), or after 10‐min of recovery (Figure 2C), between groups (all *p* > 0.05).

### 3.5 Integrated Test of Muscle Function

Peak exercise variables for the 1‐min STS test are presented in Table 2. Baseline SJ_H_ and decline in SJ_H_ was not different between groups (*p* > 0.05).

### 3.6 Body Composition

Variables for body composition are presented in Table 3. There were no significant differences between groups (all *p* > 0.05). Moreover, NWO reported elsewhere [18], was present in the group (pwCF: *n* = 4; CON: *n* = 2).

**Table 3:** Body composition data for participants with cystic fibrosis and age‐ and sex‐matched healthy controls.

Variable	CON (*n* = 14)	PwCF (*n* = 14)	*p* value	*ES*
Bone density (g·cm^‐3^)	1.05 ± 0.21	0.99 ± 0.19	0.49	0.26
FFM total (kg)	51.0 ± 16.5	48.4 ± 14.0	0.65	0.17
FFM index (kg·m^−2^)	28.8 ± 4.7	28.1 ± 3.6	0.64	0.18
FFM legs (kg)	17.2 ± 6.2	15.8 ± 4.8	0.50	0.26
FFM arms (kg)	5.7 ± 2.2	4.9 ± 1.8	0.35	0.36
FFM trunk (kg)	24.5 ± 7.7	24.1 ± 7.2	0.88	0.06
LM total (kg)	48.8 ± 15.8	46.3 ± 13.3	0.66	0.17
LM index (kg·m^−2^)	27.6 ± 4.5	26.9 ± 3.4	0.65	0.17
LM legs (kg)	16.3 ± 5.9	15.0 ± 4.5	0.50	0.26
LM arms (kg)	5.3 ± 2.1	4.6 ± 1.7	0.34	0.37
LM trunk (kg)	23.9 ± 7.5	23.5 ± 7.0	0.89	0.05
FM total (kg)	14.3 (9.8–18.1)	13.1 (11.6–20.3)	0.80	0.05
FM index (kg·m^−2^)	7.9 (6.0–10.6)	8.9 (7.2–11.0)	0.41	0.16
FM legs (kg)	5.5 ± 2.3	6.0 ± 1.8	0.59	0.21
FM arms (kg)	1.7 ± 0.7	1.8 ± 0.6	0.48	0.27
FM trunk (kg)	5.9 (3.6 – 8.1)	5.4 (4.2 – 8.0)	0.91	0.03
FM (%)	22.8 ± 7.7	24.3 ± 6.1	0.57	0.22

*Note:* Parametric values are presented as means ± SD, where data were analysed using independent *t*‐test and effect sizes were estimated using Cohen's *d*. Effect size, 0.2 = small effect, 0.5 = medium effect, 0.8 = large effect. Non‐parametric values are presented as median (interquartile range), where data were analysed using Mann−Whitney *U*‐test and effect sizes were estimated using Rosenthal's *r*. Effect size, 0.2 = weak effect, 0.4 = moderate effect, 0.6 = strong effect.

Abbreviations: CON, control group; ES, effect size; FFM, fat‐free mass; FM, fat mass; LM, lean mass; PwCF, people with cystic fibrosis.

#### 3.6.1 Relationship Between Body Composition and Muscle Strength

Peak quadriceps torque and HGS demonstrated a significant strong positive correlation with dominant leg LM and dominant arm LM, respectively, for both pwCF and CON (Figure 1).

## 4 Discussion

This study aimed to investigate comprehensively, for the first time, peripheral muscle function and body composition in pwCF stable on ETI, compared to healthy CON. Key findings were: (1) there were no differences in muscle strength, power, endurance, or fatigability between groups; (2) body composition was not different in pwCF compared to CON, and (3) a strong positive relationship was observed between muscle strength and LM, in both pwCF on ETI and CON.

Our findings are the first to show no difference in quadriceps force and HGS between pwCF on ETI and age‐ and sex‐matched healthy CON. Previous studies in pwCF, not on CFTR modulators, have reported mixed results for muscle strength, with most reporting reduced strength [3, 32, 33], while others found no differences [20]. Pre‐HEMT, reduced quadriceps force and HGS were noted [3, 33]. Whilst a major difference is the use of ETI, difference in the severity of CF lung disease compared with other studies may also contribute to these variations. A non‐significant 20% reduction in lower limb muscle strength, normalising when adjusted for limb lean mass, suggests that muscle strength deficits in pwCF on ETI may relate more to muscle mass variations than intrinsic muscle dysfunction. Our cohort also showed a strong positive correlation between LM and muscle strength, highlighting the need to consider body composition changes and identify strategies to preserve muscle mass in pwCF on HEMT who face an increased risk of sarcopenia.

We assessed quadriceps muscle endurance, by measuring the number of submaximal contractions until task failure and muscle fatigue, measured by the decline in MVC, and found no difference in muscle endurance or fatigue between the two groups. In addition, we measured muscle fatigue via the decline in SJ_H_ before versus after the 1‐min STS test and also found no difference. Whilst this is the first study to assess peripheral muscle endurance and fatigue in pwCF on ETI, studies have examined muscle endurance and fatigue in CF before HEMT. One study reported reduced endurance time during continuous isometric contraction at 50% MVC until exhaustion [34]. Another reported fewer repetitions during a functional knee bend test in females with CF compared to matched CON [35].

Our study demonstrates the number of 1‐min STS repetitions in pwCF on ETI were not different to CON. Although a significant difference in SpO_2_% was observed during the STS, the magnitude of difference (1%) is not clinically meaningful. The STS manoeuvre is frequently performed in daily life, making the STS test a good indicator of a person's ability to perform routine daily tasks [22]. Studies before HEMT have shown reduced values of 79% [36] and 71% [21], compared to predicted values. Whilst there is little evidence on the effects of HEMT on 1‐min STS performance, 1‐min STS repetitions improved significantly following 1‐year of Tezacaftor/Ivacaftor treatment [11]. A key strength of our study is the inclusion of a well‐matched control group instead of predicted values. This approach accounts for factors like location, education, equipment, protocol variations and familiarisation, which reference values cannot control for.

Our study also showed that SJ_H_, a measure of muscle power, was not different between pwCF on ETI and CON, agreeing with previous reports pre‐HEMT [35]. Little research exists on muscle power in CF, despite its importance in preventing falls in later life [37]. Although significantly reduced jump height in pwCF has been reported pre‐HEMT [38], this was not consistently observed [35]. This study is the first to use the MyJump2 application, and differences in methodology should be considered when interpreting results.

Previous studies reported complex peripheral muscle dysfunction in pwCF pre‐HEMT. Causes have included nutritional deficiency, hypoxemia, physical inactivity, corticosteroid use, and inflammation [4]. Possible intrinsic muscle defects have also been identified, including lower resting adenosine triphosphate (ATP) levels, longer phosphocreatine recovery times, and higher end‐exercise pH levels, indicating abnormal mitochondrial oxidative phosphorylation [6]. HEMT have the potential to improve muscle function through improved sarcoplasmic reticulum Ca^2+^ ATPase pump activity [9].

Studies have reported increased BMI among pwCF following ETI treatment [39]. While maintaining an optimal BMI is important, BMI alone does not reflect changes in body composition and can mask FFM depletion [15, 40]. A previous study [13] reported changes in body composition on ETI, including increases in FFM of 2.5 kg and FM of 2.1 kg. In contrast, studies with Ivacaftor report either no changes in FFM or primarily increases in FM [14, 41]. Traditionally, in pwCF FFM loss is not evenly distributed with predominant loss in the leg muscles, followed by the arms and finally the trunk. pwCF are more susceptible to lower limb muscle atrophy [40]. In our study population body composition in pwCF on ETI was not different to the control group. Specifically, we did not see FFM depletion and increases in FM or obesity as reported elsewhere. We suspect that this is a result of wide spectrum of disease severity in pwCF, as well as, the emphasis on healthy eating and regular exercise as an important component of care by the CF clinical team.

Our study was limited to a single centre and had a relatively small sample size, without pre‐ and post‐ETI data for pwCF or a control group of pwCF ineligible for ETI. The widespread availability of ETI in the UK made it impossible to carry out a study in which participants could act as their own CON. Comparing pwCF on ETI to those who are ineligible for ETI would have been confounded by many factors including varying levels of inflammation and disease severity. Although participants were matched for chronological age and sex, some matched pairs were at different stages of pubertal development. Due to the limited sample size, sub‐group analyses were not feasible. Future longitudinal research tracking changes in muscle mass and strength throughout growth and maturation would be highly valuable. Such studies are crucial for gaining deeper insights into muscle structure and function over time, particularly given the influence of pubertal development on these factors. It is possible that the exercise‐based nature of our study might have introduced a selection bias, attracting those with an interest in exercise and who had been more successful in modifying their diets after ETI treatment. Our study participants were a relatively healthy group of pwCF (mean FEV_1_: 85.7% predicted). While results might differ with a cohort having more severe disease, our data demonstrate that pwCF with good clinical outcomes can achieve muscle function comparable to healthy CON after ETI treatment. Although we did not directly control for PA levels, data from the larger Understand‐CF study suggested that there were no significant differences in PA levels between groups [18]. Further work might include replicating this study in a country where ETI is not yet available, performing measurements pre and post starting ETI. Similar studies in individuals with lower lung function might usefully clarify how ETI effects muscle function in pwCF with more severe disease.

This study is the first to show that peripheral muscle function was not different in pwCF on ETI treatment compared to a healthy control group. Further research might usefully clarify the mechanisms whereby ETI improves muscle function.

## Author Contributions

**Lauren J. Clayton:** conceptualization, methodology, data curation, investigation, writing – original draft, writing – review and editing, project administration, resources, formal analysis. **Anthony I. Shepherd:** conceptualization, investigation, funding acquisition, writing – original draft, writing – review and editing, methodology, project administration, resources, supervision, data curation. **Jo Corbett:** supervision, resources, project administration, writing – review and editing, writing – original draft, funding acquisition, conceptualization, methodology, data curation, investigation. **Mathieu Gruet:** conceptualization, writing – review and editing, methodology, writing – original draft, data curation. **Gary Connett:** conceptualization, methodology, writing – review and editingm, resourcesm, data curation. **Mark Allenby:** conceptualization, writing – review and editing, methodology, resources, data curation. **Julian Legg:** conceptualization, methodology, writing – review and editing, resources, data curation. **Thomas Daniels:** writing – review and editing, conceptualization, methodology, resources, data curation. **Don S. Urquhart:** conceptualization, methodology, writing – review and editing, writing – original draft, data curation. **Zoe L. Saynor:** conceptualization, investigation, funding acquisition, writing – original draft, writing – review and editing, methodology, project administration, resources, supervision data curation.

## Conflicts of Interest

J.L., G.C., and D.S.U. have all served as principal investigators on Vertex‐sponsored studies evaluating the use of ETI in patients with cystic fibrosis. G.C. has received speaker honoraria and research grant funding from Vertex Pharmaceuticals. D.S.U. has received speaker honoraria from Vertex Pharmaceuticals. The other authors declare no conflicts of interest.

## Supporting information

Supporting information.

## Data Availability

The data that support the findings of this study are openly available in MSK_CF_Data at https://researchportal.port.ac.uk/en/datasets/mskcfdata.
