# Supplementary material for: Peripheral Muscle Function and Body Composition in People With Cystic Fibrosis on Elexacaftor/Tezacaftor/Ivacaftor: A Cross‐Sectional Single‐Centre Study
Source: Pediatr Pulmonol. 2025 Mar 12;60(3):e71044. doi: 10.1002/ppul.71044 (PMC11898567; doi:10.1002/ppul.71044)

**Supplementary material**

**Figure S3.** Experimental set up of the quadriceps fatigue and endurance protocol and schematic of the quadriceps fatigue and endurance protocol.


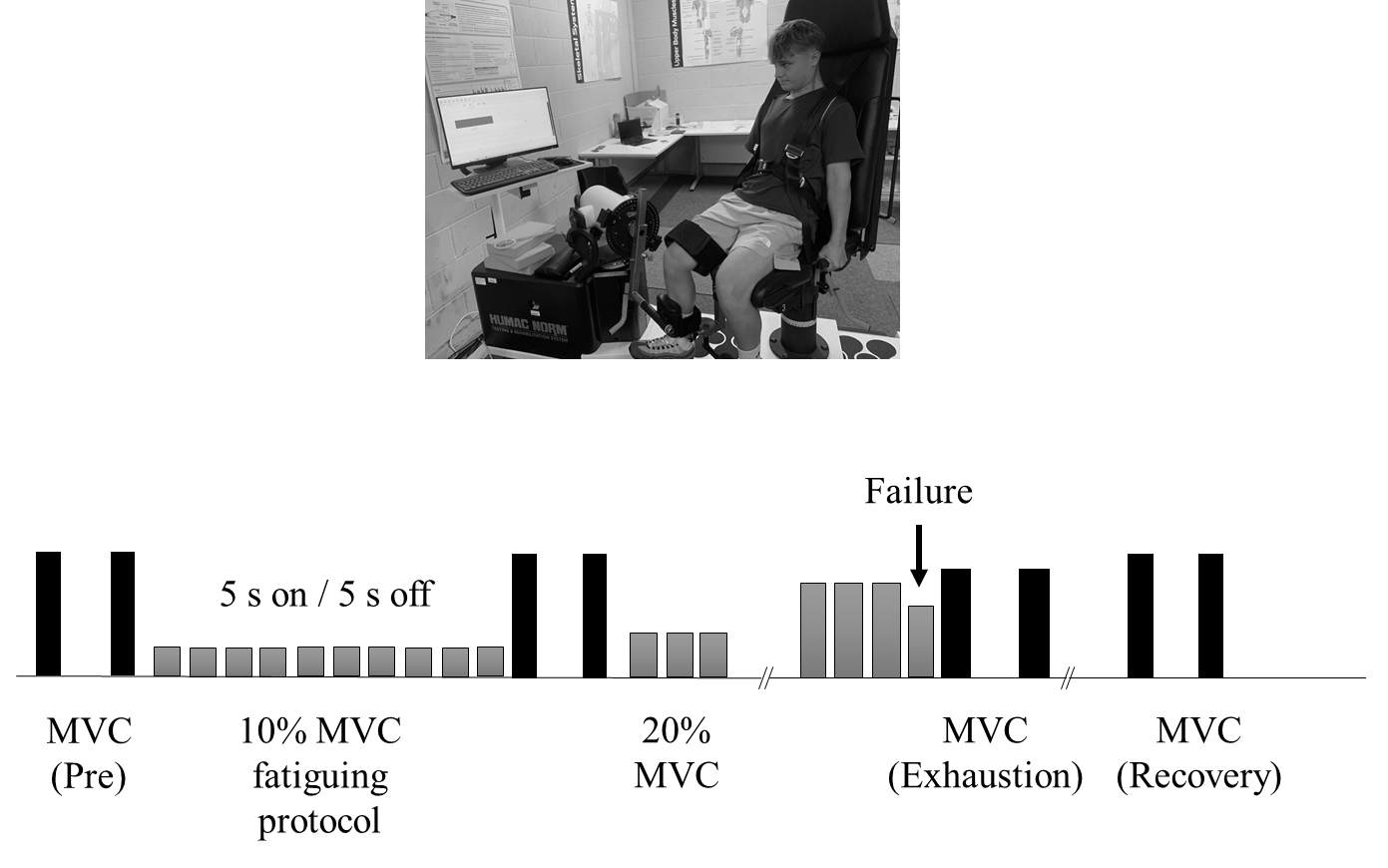


**B**

**A**

N.B. Pre-exercise maximal voluntary contraction (MVC) which was repeated after each fatiguing set. Fatiguing sets began with 10 intermittent (5-seconds on:5-seconds off) repetitions of 10% of the pre-MVC with 10% increments each set until exhaustion. Informed written consent for the use of the image included was obtained from the participant.

**Figure S4.** Schematic of squat jump and sit-to-stand protocol. Three squat jumps before and after the 1-minute sit-to-stand test


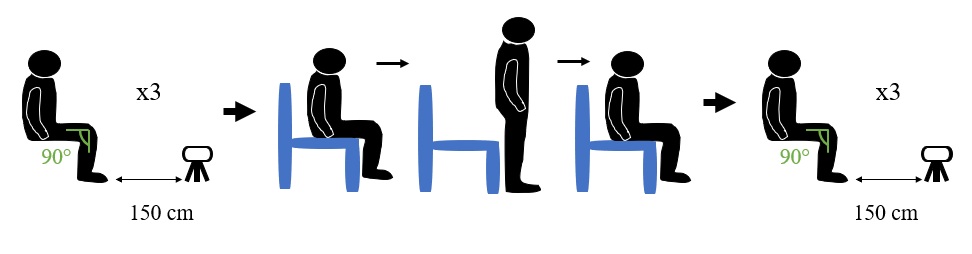

Supplement: Supplementary file 1 — Supporting information. [file PPUL-60-0-s001.docx]
